# Alpha-beta transition induced by C18-conjugation of polyalanine and its implication in aqueous solution behavior of poly(ethylene glycol)-polyalanine block copolymers

**DOI:** 10.1186/s40824-020-00200-8

**Published:** 2020-12-17

**Authors:** Min Hee Park, Jinkyung Park, Hyun Jung Lee, Byeongmoon Jeong

**Affiliations:** Department of Chemistry and Nanoscience, Ewha Womans University, 52 Ewhayeodae-gil, Seodaemun-gu, Seoul, South Korea

**Keywords:** Hydrophobic conjugation, Alpha-beta transition of polypeptide, solution behavior

## Abstract

**Background:**

The aqueous solution behavior of thermosensitive PEG-PA block copolymers as well as secondary structure of PA is expected to significantly change through modification of the hydrophobic PA by long chain alkyl (C18) groups with different configurations.

**Method:**

Oleoyl and stearoyl (C18) groups were conjugated to poly(ethylene glycol)-poly(L-alanine) (PEG-PA; EG_45_A_16_) diblock copolymers to compare their conjugation effect on nano-assemblies and corresponding aqueous solution behavior of the polymers.

**Results:**

Due to the nature of a hydrophilic PEG block and a hydrophobic PA or C18-modified PA, PEG-PA, oleoyl group-conjugated PEG-PA (PEG-PAO), and stearoyl group-conjugated PEG-PA (PEG-PAS) block copolymers form micelles in water. Compared with PEG-PA, the micelle size of PEG-PAO and PEG-PAS increased. Circular dichroism and FTIR spectra of aqueous polymer solutions showed that β sheet content increased, whereas α helix content decreased by C18 modification of PEG-PA. PEG-PAS showed better performance in ice crystallization inhibition than PEG-PAO. The sol-to-gel transition temperatures of aqueous PEG-PAO solutions were 25–37 °C higher than those of aqueous PEG-PA solutions, whereas aqueous PEG-PAS solutions remained as gels in the temperature range of 0–80 °C. ^1^H-NMR spectra indicated that the oleoyl groups increased core mobility, whereas stearoyl groups decreased the core mobility of the micelles in water. The difference in micromobility between PAO and PAS interfered or promoted gelation of the aqueous polymer solutions, respectively.

**Conclusions:**

This study suggests that a hydrophobic C18-modification of polypeptide induces α helix-to-β sheet transition of the polypeptide; however, aqueous solution behaviors including ice recrystallization inhibition and gelation are significantly affected by the nature of the hydrophobic molecule.

**Graphical abstract:**

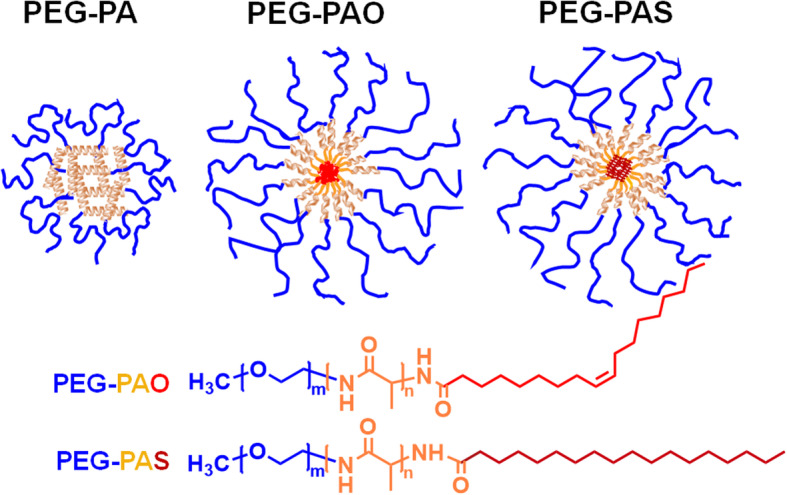

**Supplementary Information:**

The online version contains supplementary material available at 10.1186/s40824-020-00200-8.

## Background

Oleoyl and stearoyl groups are among the most common alky chains in the cellular membrane. Even though both oleoyl and stearoyl groups are C18, the structural configurations and thus biological functions are quite different due to the presence of a cis double bond at the middle of the oleoyl group. The oleoyl group has a 9,10-cis kink along the C18 alkyl chain. It is abundant in human adipose tissue as the most common unsaturated fatty acid ester [1, 2]. The oleoyl group increases flexibility of the cell membrane [3]. The biological functions such as pathogenic infection, immune competence, and materials transport across cell membranes are affected by membrane flexibility [4]. The stearoyl group comprises 30% of animal fat. The fully saturated fatty acid ester of the stearoyl group provides rigidity as well as stability of the cell membrane [5].

Aqueous solutions of poly(ethylene glycol)-poly(L-alanine) (PEG-PA) block copolymers were reported to exhibit temperature-sensitive sol-to-gel transition in a specific concentration range [6]. The aqueous solution behavior of PEG-PA as well as secondary structure of PA is expected to significantly change through modification of the hydrophobic PA by long chain alkyl (C18) groups with different configurations.

In this study, oleoyl and stearoyl groups were selected, because they have different configurations, to conjugate to the PEG-PA block copolymers. Conformational changes of the polypeptide, and aqueous solution behavior of the PEG-PA, oleoyl group-conjugated PEG-PA (PEG-PAO), and stearoyl group-conjugated PEG-PA (PEG-PAS) were compared. In particular, implication in ice recrystallization inhibition (low concentration region) and gelation behavior (high concentration region) of the aqueous polymer solutions were compared for PEG-PAO and PEG-PAS.

## Methods

### Materials

Monoamino-poly(ethylene glycol) (PEG) (M_n_ = 2000 Da) (Pharmicell, Korea) and N-carboxy anhydrides of L-alanine (Onsolution, Korea) were used as received. Oleoyl chloride and stearoyl chloride were purchased from TCI, Japan. Potassium carbonate and anhydrous N,N-dimethyl formamide were purchased from Sigma-Aldrich, USA.

### Polymer synthesis

PEG-PA was synthesized by the ring-opening polymerization of the N-carboxy anhydrides of L-alanine in the presence of monoamino-PEG [6, 7]. Polymers were purified by fractional precipitation using diethyl ether, and were dialyzed in water using a membrane with a cut-off molecular weight of 1000 Da, and freeze-dried. The final yield was 75%. The amino end group of PEG-PA (2000–1150) (2.30 g, 0.73 mmol) reacted with oleoyl chloride (0.437 g; 1.45 mmol) and stearoyl chloride (0.440 g; 1.45 mmol) in chloroform to prepare PEG-PAO and PEG-PAS, respectively. The reaction mixtures were precipitated into n-hexane, and residual solvent was removed under vacuum. The final yield was 80%.

### ^1^H-NMR spectroscopy

^1^H-NMR spectra of the polymer in CF_3_COOD (300 MHz NMR spectrometer, Bruker, USA) was used to determine the composition of the polymers. ^1^H-NMR spectra of the polymers in D_2_O (11.0 wt.%) were studied as a function of temperature to investigate the molecular mechanisms involved in sol-gel behavior.

### Gel permeation chromatography

The molecular weights and molecular weight distributions of the polymers were obtained using a gel permeation chromatography system (YL9112, Younglin, Korea) with a refractive index detector. The styragel column of HR4E (Waters, USA) was used for analysis. N,N-Dimethyl formamide was used as an eluting solvent. PEGs were used as molecular weight standards.

### Dynamic light scattering

The apparent size of PEG-PA, PEG-PAO, PEG-PAS self-assemblies were investigated by a dynamic light scattering instrument (Zetasizer Nano; Malvern Instruments Inc., USA) for aqueous polymer solutions (0.01 wt.%). A YAG DPSS-200 laser (Langen, Germany) operating at 532 nm was used as a light source.

### Transmission electron microscopy

Aqueous PEG-PA, PEG-PAO, and PEG-PAS solutions (0.01 wt.%) were placed on the 200 mesh carbon coated copper grid. The grids were dried slowly at room temperature for 24 h. The microscopic images were obtained by JEM-2100F (JEOL) with an accelerating voltage of 200 kV.

### Circular dichroism spectroscopy

Ellipticities of the PEG-PA, PEG-PAO and PEG-PAS aqueous solutions were obtained using a circular dichroism (CD) instrument (J-810, JASCO, Japan) as a function of concentration at 20 °C to infer the secondary structure of the polypeptide. The polymer concentration range varied over 0.0005*–*0.3 wt.%.

### Ice recrystallization inhibition assay and cryomicroscopy

To compare ice recrystallization inhibition (IRI) activity, each polymer solution (1.0 wt.%, 10 μl) in phosphate buffered saline (PBS) was dropped using a micropipette through a plastic tube (with a height of 1 m and a diameter of 20 cm) onto a slide glass pre-cooled on a dry ice-chilled aluminum plate [8, 9]. Upon impact, the drop instantly formed a 10 mm-diameter wafer of ice crystals on the slide glass, which was then transferred to a cryo-stage (LTS120, Linkam Scientific Instruments Ltd., UK) to be annealed at − 6 °C for 30 min. Afterwards, the cryomicroscopy images of the wafer were obtained using a microscope (CX40IT, Soptop, China) equipped with a 10X lens (UIS-2, Olympus Ltd., Japan) and a digital camera through crossed polarizers. The sizes of the ten largest crystals from each wafer were measured using the ImageJ software. The mean largest grain size was expressed as a relative size (%) obtained from PBS solution without polymers (control).

### Phase diagram

An aqueous polymer solution (0.5 mL) were placed in a test tube having an inner diameter of 11 mm. Based on the flow (sol) or no flow (gel) criterion, sol-to-gel transition temperature of the aqueous polymer solution was determined by an increment of 1 °C per step. Each data point is an average of three measurements.

### Dynamic mechanical analysis

Modulus of the aqueous polymer solutions (11.0 wt.%) were studied as a function of temperature using a rheometer (Bohlin Gemini 150; Malvern, England). The aqueous polymer solutions of PEG-PA, PEG-PAO, and PEG-PAS were placed between parallel plates with a gap of 0.5 mm and 20 mm in diameter. The modulus of the aqueous polymer solution was recorded under a controlled stress (4.0 dyne/cm^2^) with a frequency of 1.0 rad/s as a function of temperature.

### FTIR spectroscopy

The FTIR spectra (FTIR spectrophotometer FTS-800; Varian, USA) of aqueous PEG-PA, PEG-PAO, and PEG-PAS solutions (11.0 wt.% in D_2_O) were studied as a function of temperature. The aqueous polymer solutions were equilibrated for 10 min at each temperature. The amide I band at 1600*–*1700 cm^− 1^ was analyzed by the Xpeak program [10, 11].

### Statistical analysis

One-way ANOVA with Tukey tests was used for the statistical assay for ice crystal grain size. ** indicates *p* < 0.01.

## Results

**Fig. 1:**
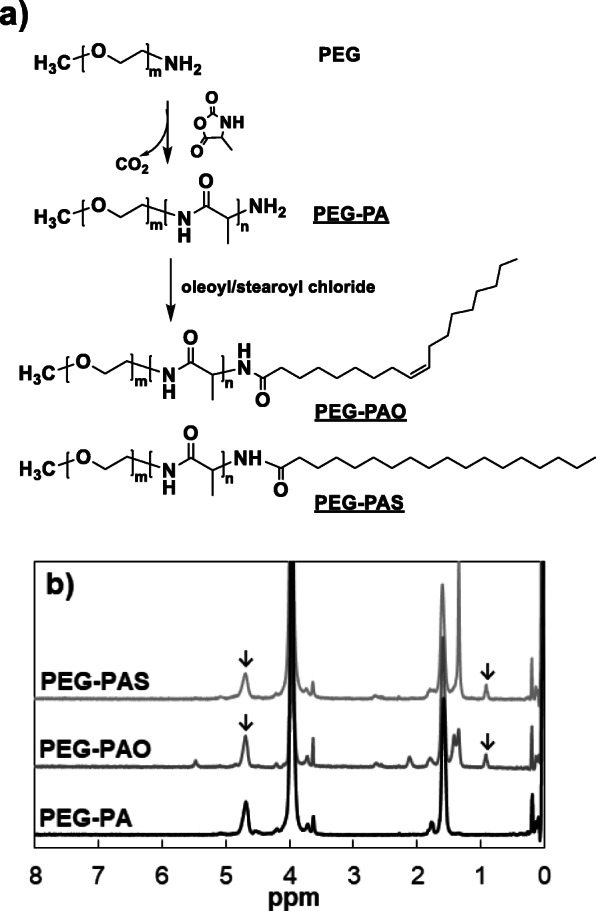
**a** Synthetic scheme of PEG-PA, PEG-PAO, and PEG-PAS. **b** ^1^H-NMR spectra of PEG-PA, PEG-PAO, and PEG-PAS. CF_3_COOD was used as an NMR solvent. m and n determined by ^1^H-NMR, are 45 and 16, respectively

The synthetic procedures of each polymer were presented (Fig. 1a). The ^1^H-NMR spectra confirmed the synthesis of PEG-PA, PEG-PAO, and PEG-PAS. The ethylene glycol peak of PEG at 3.8–4.2 ppm, methine peak of PA at 4.5–4.9 ppm, and methyl peak of oleoyl and stearoyl groups at 0.8–1.0 ppm were used to confirm the synthesis of PEG-PAO and PEG-PAS with the chemical structures of EG_45_-A_16_-oleoyl group and EG_45_-A_16_-stearoyl group, respectively (Fig. 1b). Gel permeation chromatograms of PEG-PA, PEG-PAO, and PEG-PAS exhibited peaks centered at 7.9, 8.0, and 8.0 min, respectively, indicating a similar hydrodynamic volume of the polymers in N,N-dimethyl formamide even after conjugating long chain alkyl groups to the PEG-PA. The polydispersity index (M_w_/M_n_) of PEG-PA, PEG-PAO, and PEG-PAS was 1.2, 1.1, and 1.1, respectively.

**Fig. 2:**
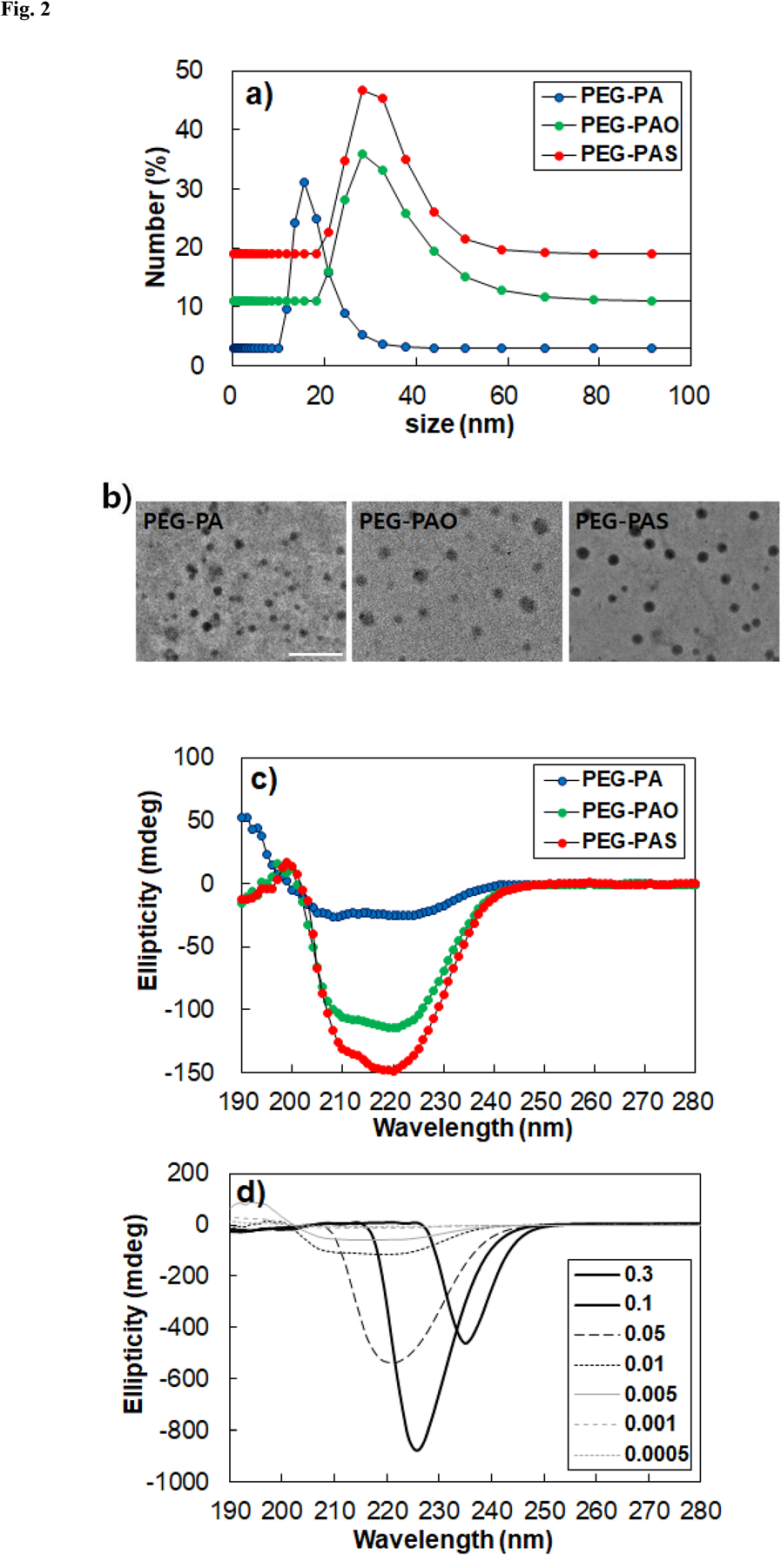
**a** Apparent size of PEG-PA, PEG-PAO, and PEG-PAS aqueous solutions (0.01 wt.%) at 20 °C. **b** Transmission electron microscopy images of PEG-PA, PEG-PAO, and PEG-PAS micelles developed from their aqueous solution (0.01 wt.%). The scale bar is 100 nm. **c** CD spectra of PEG-PA, PEG-PAO, and PEG-PAS aqueous solutions (0.01 wt.%) at 20 °C. **d**) CD spectra of aqueous PEG-PAO as a function of concentration at 20 °C. The legends are concentration (wt.%) of polymers in water

Apparent sizes of the micelles were provided by dynamic light scattering of aqueous PEG-PA, PEG-PAO, and PEG-PAS solutions (0.01 wt.%), where the peak average micelle sizes were 16, 28, and 28 nm, respectively (Fig. 2a). The transmission electron microscope images developed from their aqueous polymer solutions (0.01 wt.%) exhibit the spherical micelle structures of PEG-PA, PEG-PAO, and PEG-PAS (Fig. 2b). CD spectra of the aqueous polymer solutions (0.01 wt.%) were compared at 20 °C. PEG-PA exhibited the typical α helix with two minima at 210 and 223 nm. A significant increase in the magnitude of ellipticity of the polymers was noticeable for PEG-PAO and PEG-PAS, compared with PEG-PA (Fig. 2c). The ellipticities at 210 nm were − 25 to − 105 and − 131 mdeg for aqueous PEG-PA, PEG-PAO, and PEG-PAS solutions, respectively. The large differences come from changes in secondary structure as well as aggregation of the polypeptides. As the concentration of the polymer increased from 0.0005 to 0.3 wt.%, red shifts of the CD spectra were observed for aqueous PEG-PA, PEG-PAO, and PEG-PAS solutions, and the CD spectra developed a single large band. Micelle formation of the polypeptide-containing polymers leads to the red shift in the CD spectra [10, 12]. In the case of PEG-PAO, the band shifted to 221 nm (0.05 wt.%), 226 nm (0.1 wt.%), and 235 nm (0.3 wt.%) (Fig. 2d). PEG-PA and PEG-PAS exhibited similar trends (Supplementary Information: Fig. S1).

**Fig. 3:**
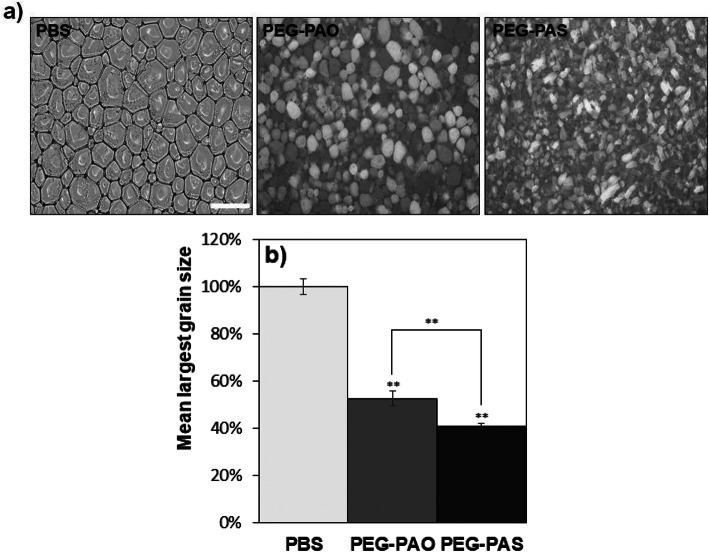
Ice recrystallization inhibition effect of PEG-PAO and PEG-PAS aqueous solutions (1.0 wt.%). The microscopy images of the ice crystals (**a**) and mean largest grain size (**b**) were compared. The scale bar is 200 μm. ** on the bar graph indicates *p* < 0.01 in comparison with PBS

Differences in self-assemblies of PEG-PA, PEG-PAO, and PEG-PAS were demonstrated in the ice recrystallization inhibition (IRI) activity. Phosphate buffered saline (PBS) developed ice crystals of about 100 μm in size. The addition of PEG-PAO and PEG-PAS to PBS decreased the size of the ice crystals of the solution (1.0 wt.%) (Fig. 3a). The mean largest grain size decreased to 53 and 41% of that of the PBS (control) for PEG-PAO and PEG-PAS solutions, respectively, indicating that PEG-PAS are statistically (*p* < 0.01) more effective than PEG-PAO in IRI activity (Fig. 3b).

**Fig. 4:**
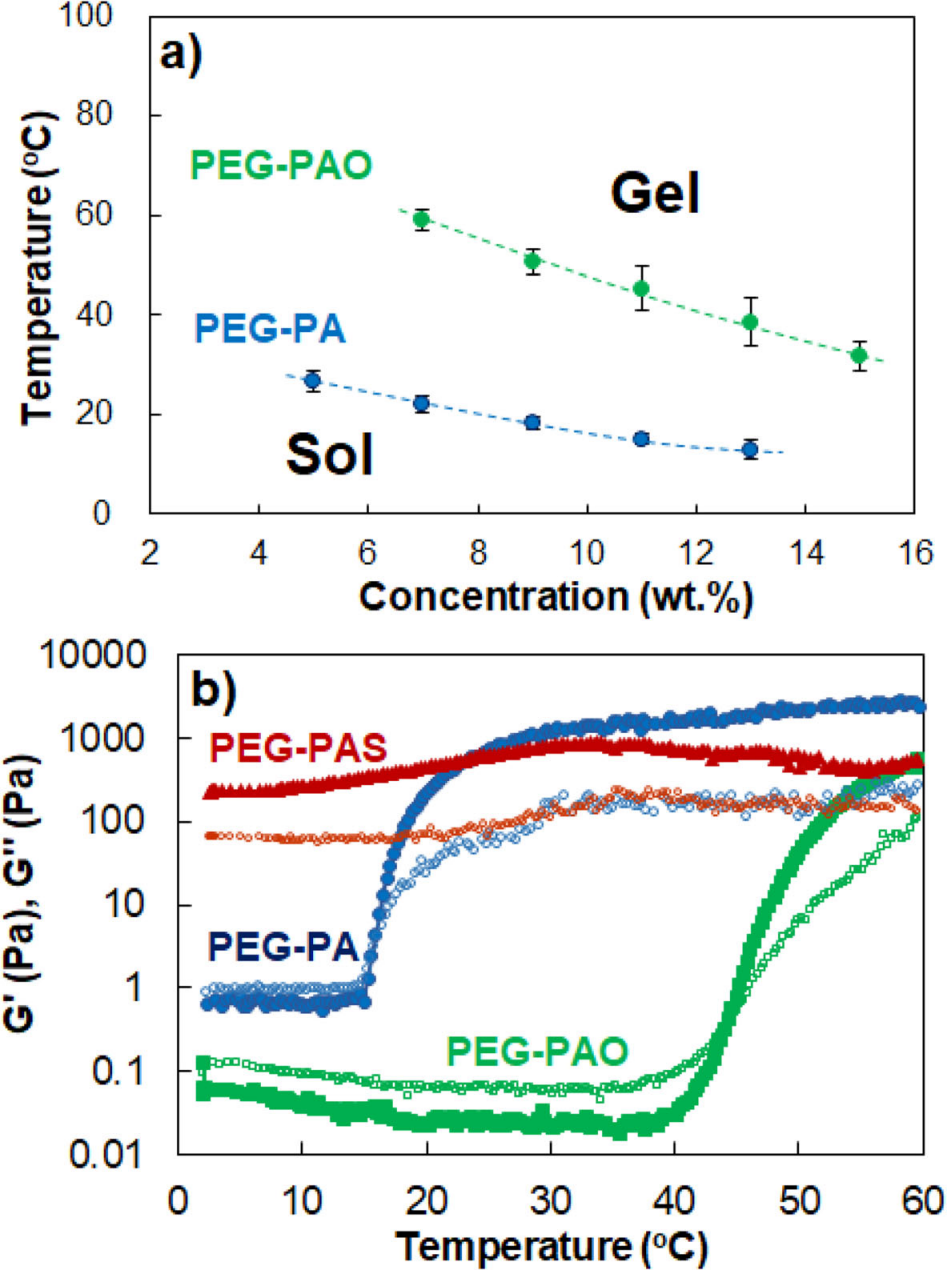
**a** Phase diagram of PEG-PA, PEG-PAO, and PEG-PAS aqueous solutions. **b** Storage modulus (G’: filled large figures) and loss modulus (G”: empty small figures) of PEG-PA, PEG-PAO, and PEG-PAS aqueous solutions (11.0 wt.%) as a function of temperature

Significant differences were observed among aqueous PEG-PA, PEG-PAO, and PEG-PAS solutions in sol-gel transition behavior at high concentration region of the polymers (Fig. 4a). Aqueous PEG-PA solutions undergo sol-to-gel transition as the temperature increased in a concentration range of 5.0*–*13.0 wt.%. Aqueous PEG-PAO solutions also showed a similar sol-to-gel transition in a concentration range of 7.0*–*15.0 wt.%. However, the transition temperatures increased by 25*–*37 °C over the same concentration regions. On the other hand, PEG-PAS showed a gel phase over the same concentration range and did not show sol-to-gel transition in the investigated temperature range of 0*–*80 °C. Moduli of aqueous PEG-PA, PEG-PAO, and PEG-PAS solutions (11.0 wt.%) were investigated as the temperature increased (Fig. 4b). Storage modulus (G’) and loss modulus (G”) are the elastic component and viscous component of complex modulus, respectively. Therefore, crossing of G’ over G” indicates sol-to-gel transition [13]. The crossing point of G’ over G” was observed at 16 and 46 °C for aqueous PEG-PA and PEG-PAO solutions, respectively. However, G’ of PEG-PAS was greater than G” in the same temperature range, indicating a gel phase persisted without sol-gel transition.

**Fig. 5:**
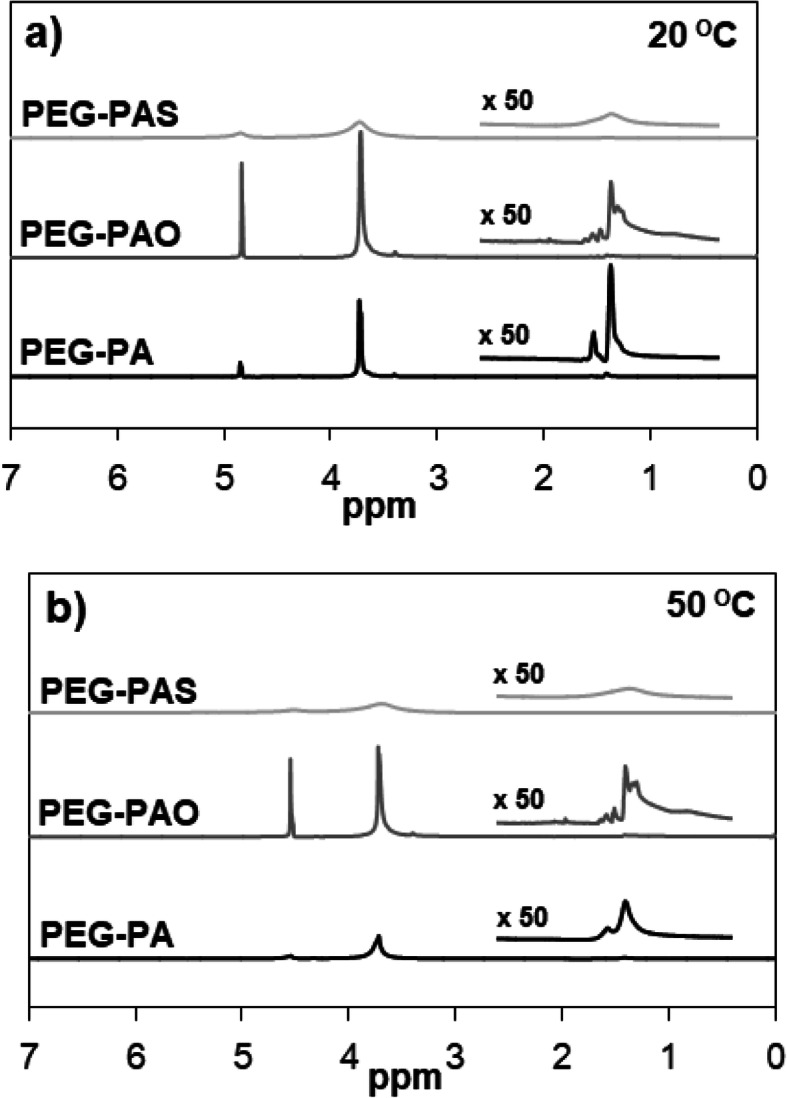
^1^H-NMR spectra of PEG-PA, PEG-PAO, and PEG-PAS in D_2_O at 20 °C (**a**) and 50 °C (**b**)

**Fig. 6:**
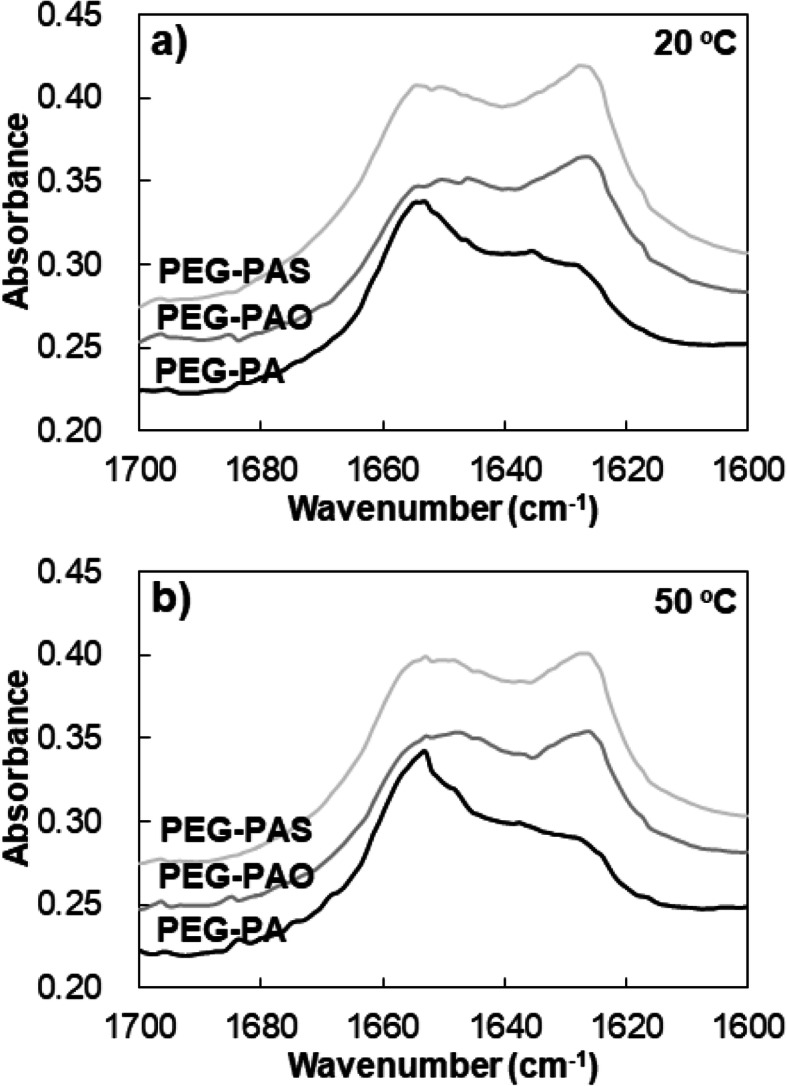
FTIR spectra of PEG-PA (**a**), PEG-PAO (**b**), and PEG-PAS (**c**) aqueous solutions (11.0 wt.%) as a function of temperature

In the search for a mechanism of the difference in the molecular level, ^1^H-NMR and FTIR spectra of aqueous polymer solutions (11.0 wt.% in D_2_O) were compared at 20 and 50 °C (Figs. 5 and 6). The peaks at 3.3*–*3.9 ppm (PEG) and 4.2*–*5.0 ppm (D_2_O) were sharp for PEG-PAO (sol phase), whereas they slightly collapsed for PEG-PA (just above sol-to-gel transition) at 20 °C. The peaks at 1.0*–*2.0 ppm (PA or PAO) were significantly collapsed at 20 °C due to the core-shell nature of the polymers. The peaks corresponding to hydrophobic blocks tend to collapse in the ^1^H-NMR spectra using D_2_O because they located in the core of micelles [14, 15]. In particular, the PAO peaks (1.0*–*2.0 ppm) were more collapsed than the PA peaks (1.0*–*2.0 ppm) at 20 °C, whereas PA peaks were more collapsed than the PAO peaks at 50 °C. On the other hand, the PEG peak at 3.3*–*3.9 ppm was sharp for PEG-PAO (sol phase), however it was collapsed for PEG-PA (just above sol-to-gel transition) at 20 °C. The PEG peak was collapsed for PEG-PAO (just above sol-to-gel transition) and significantly collapsed for PEG-PA (gel phase) at 50 °C.

The FTIR spectra of the aqueous polymer solution (11.0 wt.% in D_2_O) were also studied to compare changes in secondary structures of the polypeptides. The bands at 1620*–*1630 cm^− 1^ and 1650*–*1660 cm^− 1^ are attributed to β sheet and α helix conformation of the polypeptide, respectively [16, 17]. As demonstrated in the FTIR spectra, the conjugation of hydrophobic alkyl chains of the oleoyl group or stearoyl group to PEG-PA, the β sheet bands at 1620*–*1630 cm^− 1^ increased at both 20 and 50 °C (Fig. 6 and Supplementary Information: Fig. S2). Amide I bands in 1600*–*1700 cm^− 1^ were analyzed by Xpeak software for semi-quantitative assay of the secondary structure [10, 11]. Xpeak analysis indicated that α helix content decreased from 48% to 33*–*34%, whereas the β sheet content increased from 31 to 44*–*45% by C18 conjugation (Table 1).

**Table 1:** Analysis of secondary structures of polypeptide by using Xpeak software

polymer	α helix/β sheet/ random coil (20 °C)	α helix/β sheet/ random coil (50 °C)
PEG-PA	48/31/21	46/28/26
PEG-PAO	34/44/22	33/39/28
PEG-PAS	33/45/21	32/41/27

β sheet: 1625 and 1633 cm^− 1^, random coil: 1646 cm^− 1^ [8, 9]

## Discussion

PEG-PA was synthesized by ring-opening polymerization of N-carboxy anhydrides of L-alanine in the presence of monoamino-PEG [6, 7]. About two times excess amount of oleoyl chloride and stearoyl chloride reacted with the amino-end group of PEG-PA to prepare PEG-PAO and PEG-PAS, respectively, which led to the polymers without contamination of unreacted PEG-PA. ^1^H-NMR and GPC confirm the correct structure of PEG-PA, PEG-PAO and PEG-PAS.

Due to the hydrophilic block (PEG) and hydrophobic block (PA, PAO and PAS), the PEG-PA, PEG-PAO, and PEG-PAS block copolymers self-assembled in water. Micelles are expected to form in water due to the large ratio of the hydrophilic block to hydrophobic block for all of the PEG-PA, PEG-PAO, and PEG-PAS block copolymers [18]. Increases in the micelle size of PEG-PAO and PEG-PAS in water might be the result of the increased hydrophobic aggregation of the polymers in the core of a micelle after conjugating the long alkyl chain to PEG-PA (Fig. 2a). There might be some distortion of the micelle structures during the air-drying process in the transmission electron microscope measurements, however, the spherical shape of the micelles were clearly observed in the images (Fig. 2b).

CD spectra of the aqueous polymer solutions suggested that the β sheet content of PEG-PA increased after conjugating the long hydrophobic alkyl chain to PEG-PA. It is notable that the α helix-to-β sheet transition of polypeptides could be induced by hydrophobic modification of the polypeptide.

Ice recrystallization inhibition (IRI) activity is very important in cell storage or food industry [8, 9, 19]. IRI activity of PEG-PA, PEG-PAO, and PEG-PAS were compared. Ice crystals have rather hydrophobic surfaces and adhesion of amphiphilic molecules tends to inhibit growth of ice crystals [20]. Therefore, the control of hydrophobic and hydrophilic balance is important to regulate IRI activity. PEG-PAS are more effective than PEG-PAO in IRI activity (Fig. 3b). PEGs on the ice crystal surface interfere approaching of water molecules that need for growth of ice crystals. The PEG population per unit area of PEG-PAS-coated ice crystal can be higher than that of PEG-PAO-coated ice crystal due to the larger free volume of an oleoyl group with a cis kink at 9,10-position along C18 alky chain. Therefore, PEG-PAS could be more effective than PEG-PAO in IRI activity.

The sol-gel transition behavior were significantly different among aqueous PEG-PA, PEG-PAO, and PEG-PAS solutions (Fig. 4a). The increase in sol-to-gel transition temperature of an aqueous PEG-PAO solution suggests that interference in the sol-to-gel transition occurs by conjugation of the oleoyl group to PEG-PA even though hydrophobicity of the polymer increases. Usually, the sol-to-gel transition of aqueous polymer solutions is facilitated by an increase in hydrophobicity of the polymer [21–25]. On the other hand, PEG-PAS showed a gel phase over the same concentration and temperature ranges, indicating gel phase is stabilized for PEG-PAS.

Based on ^1^H-NMR and FTIR spectra of aqueous polymer solutions (11.0 wt.% in D_2_O), the mechanism of the difference in the molecular level were suggested. ^1^H-NMR spectra suggested that molecular motion of PEG significantly decreased as the temperature increased from 20 to 50 °C, whereas oleoyl groups maintained their molecular motion over 20*–*50 °C. The flexibility of the conjugated oleoyl group of PEG-PAO might be responsible for interference of thermogelation of the aqueous PEG-PAO solution, compared with the aqueous PEG-PA solution. The aqueous PEG-PAS solutions (11.0 wt.%) remained as a gel phase and all peaks of PEG-PAS were significantly collapsed at 20 and 50 °C. Similar to their roles in the cell membrane, the oleoyl group with a cis-kink at the middle of the long alkyl chain might provide fluidity or flexibility of the core with the core-shell structure of PEG-PAO assemblies, and interferes with thermogelation of the aqueous PEG-PAO solution. Whereas the stearoyl group with saturated alkyl chain might provide rigidity of the core with core-shell structure of PEG-PAS assemblies, and facilitated gel formation of the aqueous PEG-PAS solution. The significance of core-softness related to sol-gel transition of the aqueous polymer solutions was also manifested in aqueous solutions of poly(ethylene glycol)-poly(L-alanine) (PEG-L-PA) vs poly(ethylene glycol)-poly(DL-alanine) (PEG-DL-PA), poly(L-lactic acid)-poly(ethylene glycol)-poly(L-lactic acid) (L-PLA-PEG-L-PLA) vs poly(DL-lactic acid)-poly(ethylene glycol)-poly(DL-lactic acid) (DL-PLA-PEG-DL-PLA), and the stereocomplex of L-PLA-PEG-L-PLA and poly(D-lactic acid)-poly(ethylene glycol)-poly(D-lactic acid) (D-PLA-PEO-D-PLA) [26–29]. All polymers consist of hydrophilic PEG and hydrophobic poly(alanine) or poly(lactic acid), therefore the block copolymers formed core-shell micellar structure in water. However, the sol-gel transition behavior was quite different due to the core softness of the self-assemblies. For example, sol-to-gel transition temperatures of aqueous PEG-DL-PA (1000–780) solutions were 60*–*80 °C in a concentration range of 16*–*24 wt.%, whereas those of the aqueous PEG-L-PA (1000–780) solutions were 10*–*40 °C in a concentration range of 6*–*12 wt.% [26]. DL-PAs develop soft random coil structures, whereas L-PAs develop rigid β sheet or α helix structures. Similarly, DL-PLAs develop a soft core structures, whereas L-PLAs and the stereocomplex of L-PLA/D-PLA developed a stiff core of the polymer self-assemblies, and thus facilitate gelation of their aqueous solutions [27–29].

As discussed in CD spectra, α helix-to-β sheet transition by hydrophobic conjugation to the polypeptide was noticeable in FTIR spectra. The secondary structures of polypeptide for PEG-PA, PEG-PAO, and PEG-PAS were basically maintained when the temperature increased from 20 to 50 °C except for partial decreases in β-sheet content and increases in random coil content. The FTIR spectra suggested that the long chain alky (C18) group conjugated to PEG-PA significantly promoted β sheet structures of PA by attractive forces in water for hydrophobic aggregation of the chain ends. Modifications of poly(L-alanine)-poloxamer-poly(L-alanine) (PA-PLX-PA) by short alkyl groups including methyl, ethyl, propyl, and butyl groups increased β-sheet content of PA through hydrophobic interactions [30]. Contrary to current C18-modifications which increased sol-to-gel transition temperature (PEG-PAO) or eliminated of sol-to-gel transition (PEG-PAS), the short alky group modifications steadily decreased sol-to-gel transition temperature of the aqueous polymer solutions as the hydrophobicity of the polymer increased. The differences in sol-gel transition behavior induced by the short chain and long chain alkyl group modifications suggest that different mechanisms such as strong hydrophobic interactions and conformational degree of freedom of the long alkyl (C18) chains as well as changes in secondary structures of PA might be involved. The different mechanisms among the short alky group and long alkyl groups are also well-known for the glass transition temperature of polyacrylates [31]. Glass transition temperature of polyacrylates exhibits a minimum curve as the alky group length increases. Plasticizing effects of short alkyl (C1-C8) groups decrease glass transition temperature of the polyacrylates as the alkyl chain length increases, whereas strong intermolecular interactions among the long alkyl (C12-C16) groups overwhelm the plasticizing effects and increase the glass transition temperature of the polyacrylates as the alkyl chain length increases. Previously, we reported that the β sheet content of the polypeptide was reduced by hydrophilic PEG conjugation to an oligopeptide, penta-alanine (A_7_) [6]. As the molecular weight of PEG increased from 1000 to 2000, and 5000 Da, the β sheet content of PEG-A_7_ decreased from 56% to 45 and 33%, respectively. PEG is in a highly dynamic motion in water, which might interfere with the unique molecular arrangements leading to β sheet conformation of the polypeptide. Conformation of polypeptides such as the α helix, β sheet, and random coil plays a significant role in determining structures as well as biological activities of the polypeptides and proteins [32]. In particular, α helix-to-β sheet transition of polypeptides is reported to be involved in Creutzfeldt-Jakob and Alzheimer’s diseases [33, 34]. The transition proceeds by unfolding of α helix followed by rearrangements to β sheet formation, which leads to aggregation of the polypeptides and sometimes results in precipitation of the polypeptides. However, the molecular structure that triggers α helix-to-β sheet transition of polypeptides or proteins are yet clear. Here we suggest that conjugating the hydrophobic group to a polypeptide induces α helix-to-β sheet transition of the polypeptide, whereas conjugating the hydrophilic PEG to a polypeptide reduces β sheet content of the polypeptide.

**Fig. 7:**
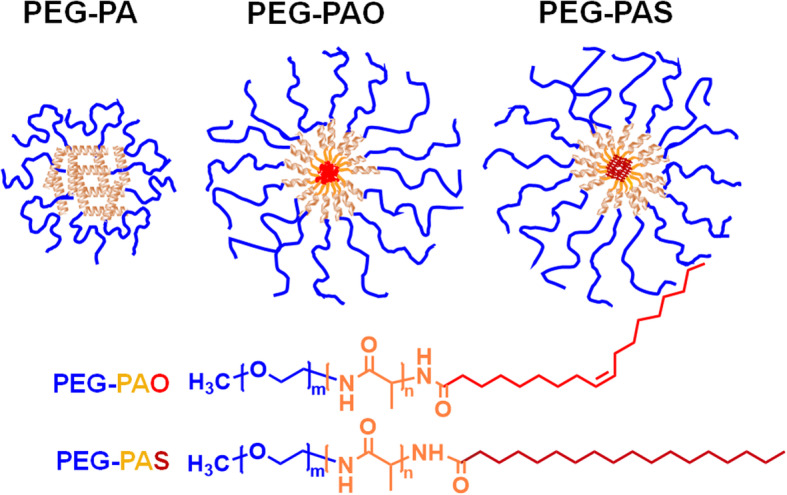
Schematic presentation of PEG-PA, PEG-PAO, and PEG-PAS self-assemblies in water. Blue, gold, red, and dark red curves indicate PEG, PA, oleoyl group, and stearoyl group, respectively. m and n are 45 and 16, respectively. C18 modification of PA induces conformational transition of PA from α helix-to-β sheet and increases micelle size of the polymers in water. Oleoyl and stearoyl groups provide rigidity and flexibility, respectively, of the core of the micelles. Therefore, the oleoyl group interferes with gelation, whereas the stearoyl group facilitates gelation

Based on the above results, the effects of the oleoyl group and stearoyl group conjugation to PEG-PA are schematically presented (Fig. 7). Blue, gold, red, and dark red curves indicate PEG, PA, oleoyl group, and stearoyl group, respectively. First, PEG-PA, PEG-PAO, PEG-PAS form micelles in water due to their amphiphilic nature consisting of a hydrophilic block (PEG) and a hydrophobic block (PA, PAO, and PAS). C18 modification of PA not only increases aggregation tendency or micelle size of the polymers in water but also increases β sheet content of the PA. On the other hand, oleoyl and stearoyl groups affect rigidity/flexibility of the core of the micelles, thus affect the solution behavior of the polymers.

## Conclusions

C18-modified PEG-PA block copolymers were synthesized to investigate how the configuration difference in C18 affects the solution behavior of PEG-PA block copolymers. C18 modification increases the micelle size and induced α helix-to-β sheet transition of the PA through the increased hydrophobic aggregation of the polypeptides. IRI activity of PEG-PAS was higher than that of PEG-PAO. PEG-PAO with the oleoyl group interfered with gelation and increased sol-to-gel transition temperature. However, PEG-PAS with the stearoyl group facilitated the gelation of the aqueous polymer solutions. The large free volume of the oleoyl group with a cis kink in the middle of the C18 chain and core flexibility of the micelles provided by the oleoyl groups might be responsible for such behavior. To conclude, the hydrophobic modification of the PEG-PA block copolymer affects the secondary structure of polypeptide (PA), and the solution behavior also seriously altered by the configuration of the hydrophobic molecule.

## Supplementary Information

**Additional file 1.**

## Data Availability

For data requests, please contact the authors.
