# Supplementary material for: Alpha-beta transition induced by C18-conjugation of polyalanine and its implication in aqueous solution behavior of poly(ethylene glycol)-polyalanine block copolymers
Source: Biomater Res. 2020 Dec 17;24:23. doi: 10.1186/s40824-020-00200-8 (PMC7745361; doi:10.1186/s40824-020-00200-8)

**Supplementary Information**

Alpha-beta transition induced by C18-conjugation of polyalanine and its implication in aqueous solution behavior of poly(ethylene glycol)-polyalanine block copolymers

Min Hee Park, Jinkyung Park, Hyun Jung Lee, and Byeongmoon Jeong*

Department of Chemistry and Nanoscience, Ewha Womans University, 52 Ewhayeodae-gil, Seodaemun-gu, Seoul, Korea

* Corresponding author.

E-mail addresses: [bjeong@ewha.ac.kr](mailto:bjeong@ewha.ac.kr), Tel.: +82 2 3277 3411; Fax: +82 2 3277 3419

**Fig. S1.** CD spectra of PEG-PA (a) and PEG-PAS (b) aqueous solutions as a function of polymer concentration at 20 ^o^C. Legends are polymer concentrations in wt.% in water.


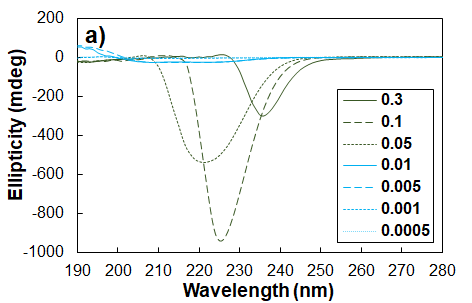


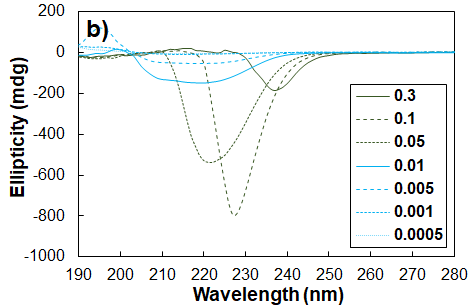


**Fig. S2.** FTIR spectra of PEG-PA (a), PEG-PAO (b), and PEG-PAS (c) in D_2_O (11.0 wt.%) as a function of temperature. The legends are temperature in ^o^C.


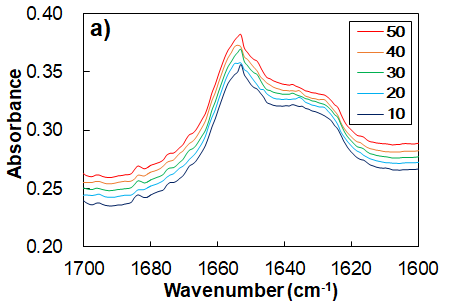


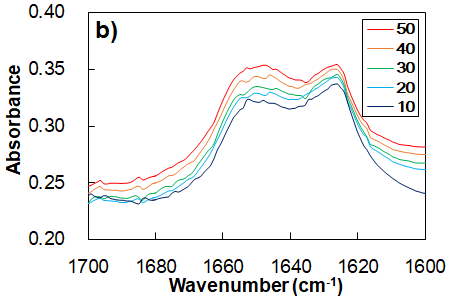

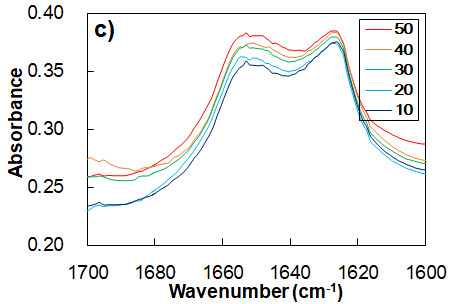

Supplement: Supplementary file 1 — Additional file 1. [file 40824_2020_200_MOESM1_ESM.doc]
